# Relation between Coronary Artery Calcium Score and Cardiovascular Events in Hodgkin Lymphoma Survivors: A Cross-Sectional Matched Cohort Study

**DOI:** 10.3390/cancers15245831

**Published:** 2023-12-13

**Authors:** Elissa A. S. Polomski, Julius C. Heemelaar, Michiel A. de Graaf, Augustinus D. G. Krol, Marloes Louwerens, J. Lauran Stöger, Paul R. M. van Dijkman, Martin J. Schalij, J. Wouter Jukema, M. Louisa Antoni

**Affiliations:** 1 Department of Cardiology, Heart Lung Center, Leiden University Medical Center, 2333 ZA Leiden, The Netherlands; 2 Department of Radiotherapy, Leiden University Medical Center, 2333 ZA Leiden, The Netherlands; 3 Department of Internal Medicine, Leiden University Medical Center, 2333 ZA Leiden, The Netherlands; 4 Department of Radiology, Leiden University Medical Center, 2333 ZA Leiden, The Netherlands; 5 Netherlands Heart Institute, 3511 EP Utrecht, The Netherlands

**Keywords:** Hodgkin lymphoma, radiotherapy, coronary artery calcium score, coronary artery disease

## Abstract

**Simple Summary:**

This study compares the presence of coronary artery calcium on coronary computed tomography angiography in relation to cardiovascular events between Hodgkin lymphoma (HL) survivors treated with thoracic radiotherapy and a matched non-cancer control group. HL survivors have a higher prevalence of coronary artery calcium more than ten years after irradiation. However, HL patients with a coronary artery calcium score of zero still have an increased risk of future cardiovascular events, possibly due to rapid progression of atherosclerosis in the coronary arteries following irradiation. Timely treatment with statins should be considered to prevent rapid acceleration of pre-existing atherosclerosis.

**Abstract:**

Background: Thoracic radiotherapy is one of the corner stones of HL treatment, but it is associated with increased risk of cardiovascular events. As HL is often diagnosed at a young age, long-term follow-up including screening for coronary artery disease (CAD) is recommended. Objectives: This study aims to evaluate the presence of coronary artery calcium score (CACS) in relation to cardiovascular events in HL patients treated with thoracic radiotherapy compared to a non-cancer control group. Methods: Consecutive HL patients who underwent evaluation for asymptomatic CAD with coronary computed tomography angiography > 10 years after thoracic irradiation were included. The study population consisted of 97 HL patients matched to 97 non-cancer patients on gender, age, cardiovascular risk factors, and statin use. Results: Mean age during CT scan in the HL population was 45.5 ± 9.9 and in the non-cancer population 45.5 ± 10.3 years. CACS was elevated (defined as >0) in 49 (50.5%) HL patients and 30 (30.9%) control patients. HL survivors had an odds ratio of 2.28 [95% CI: 1.22–4.28] for having a CACS > 0 compared to the matched population (*p* = 0.006). Prevalence of CACS > 90th percentile differed significantly: 17.1% in HL survivors vs. 4.6% in the matched population (*p =* 0.009). Non-obstructive coronary artery stenosis was more prevalent in the HL population than in the control population (45.7% vs. 28.4%, respectively, *p* = 0.01). During follow-up of 8.5 [5.3; 9.9] years, nine HL patients experienced an event including two patients with a CACS of zero. No events occurred in the control population. Conclusion: In a matched study population, HL survivors have a higher prevalence of a CACS > 0 and an increased risk of cardiovascular events after thoracic irradiation compared to a matched non-cancer control group.

## 1. Introduction

Treatment for Hodgkin lymphoma (HL) has improved significantly, leading to better long-term prognosis and a 10-year survival of >80% [1]. Thoracic radiotherapy and chemotherapy are widely used therapeutic regimens in the treatment of HL. However, both have been shown to increase the risk of cardiovascular events [2,3,4]. Radiotherapy can cause cardiac damage at the microvascular and macrovascular level, leading to coronary artery disease (CAD) as an important long-term complication [5]. Especially in the young population of HL survivors with overall good prognosis after treatment, this can lead to significant morbidity and mortality.

Compared to the general population, CAD after radiation presents more often with ostial lesions and multivessel disease [6]. In addition, plaque composition seems to be different as small studies have shown increased calcium scores, as well as soft plaques leading to significant stenosis, while screening asymptomatic HL patients treated with thoracic irradiation [7,8]. Human pathology studies have described that intimal lesions following radiation exposure consist primarily of fibrous tissue, while a minority of lesions contain lipid or calcium deposits in addition to fibrosis [9].

Current guidelines recommend to start non-invasive screening for CAD in asymptomatic patients who received >15 Gy mean heart dose (MHD) or ≥35 Gy radiation to a volume exposing the heart at 5–10-year intervals, starting at 5 years after irradiation [10]. Therefore, anatomical imaging using coronary artery calcium (CAC) and coronary computed tomography angiography (CCTA) may play a key role in this population. CAC can be scored binary to indicate the presence of atherosclerosis or with an Agatston score to assess the extent of calcification in the coronary arteries.

In the general population, a coronary artery calcium score (CACS) of zero progresses to a CACS > 10 in approximately 5–8 years. However, progression within 10 years to a CACS > 100 is rarely observed [11]. Research in large study populations with CAC scores of zero has shown that, after a follow-up period of 10 years, only 4% of the patients experienced a cardiovascular event and that asymptomatic individuals have mortality-free survival period of 15 years [12]. Since the natural history of radiation-induced vasculopathy is different from atherosclerosis and may accelerate rapidly, different monitoring intervals may be needed in this specific patient group.

Therefore, this study aims to evaluate the presence and distribution of CAC and the stenosis severity in relation to cardiovascular events in asymptomatic HL patients treated with thoracic radiotherapy compared to a matched non-cancer control group.

## 2. Methods

### 2.1. Study Population

This study was conducted as a cross-sectional matched cohort study. Consecutive HL patients who were referred to the outpatient cardio-oncology clinic at the Leiden University Medical Center (LUMC) as part of the Dutch National Survivorship Care Pathway [13] were assessed for eligibility. Patients were included if they had been treated with radiotherapy alone, or in combination with other cancer therapies, and if they were referred for CCTA > 10 years after radiotherapeutic treatment. All HL patients were asymptomatic and CCTA was performed as cardiovascular screening method. The HL patients were matched 1:1 to a control group consisting of symptomatic non-cancer patients who were referred for CCTA at the LUMC for evaluation of chest pain. Patients were matched on gender, age (±5 years), hypertension, hypercholesterolemia, smoking, and statin use. Patient history and cardiovascular risk factors of matched patients were verified through manual chart review. Hypercholesterolemia was defined as a history of hypercholesterolemia or a total cholesterol > 5 mmol/L at baseline visit. Smoking was defined as a history of smoking or active smoking. Patients with prior acute coronary syndrome (ACS) or coronary artery bypass grafting (CABG) were excluded from the study population. Informed consent was obtained from all patients for participation in this study. The study was approved by the local Medical Ethical Committee (METC-LDD G20.045) and complies with the declaration of Helsinki.

### 2.2. Data Collection

Demographic patient data, laboratory results, medication, cardiac CT reports, echo reports, and cardiovascular risk factors were collected through the departmental cardiology information system (EPD-Vision^®^, Denmark, The Netherlands) and pharmacy records (HiX Version 6.1 Chipsoft, Amsterdam, The Netherlands). Echocardiographic data were collected for baseline echocardiogram, defined as closest to date of cardiac CT scan. Demographic patient data, medication, and cardiovascular risk factors of the matched control group had previously been collected in an institutional database consisting of 7000 patients who underwent CCTA at the LUMC. These data were manually checked for correctness after the matching procedure. Data on oncological characteristics, including the date of HL diagnosis and detailed information on radiation and chemotherapy, were collected from the internal oncology registry (OncDoc), which is connected to the Netherlands Cancer Registry.

### 2.3. CCTA Acquisition

All patients were scanned using a 320-row volumetric scanner (Acquillon ONE Canon Medical Systems, Otawara, Japan). A single dose metoprolol was administered orally (25–150 mg) 60 min prior to the examination if the heart rate was >65 beats per minute and no contra-indications were present. If the target heart rate was not achieved after 60 min, metoprolol was administered intravenously (2.5–10 mg). Sublingual nitroglycerin spray was given five minutes prior to examination. Scan parameters were set according to body mass index (BMI). Tube voltage ranged between 80 and 135 kV, tube current was set between 190 and 900 mA, gantry rotation time was 275–350 ms, and the scan range chosen differed between 12 and 16 cm. Contrast administration was also dependent on BMI and ranged between 50 and 95 mL, which was administered in 11 s (FlowSens Medes^®^, Languedoc-Roussillon, France) and followed by a 30–60 mL saline flush at 10 s. To reduce radiation dose, prospective ECG-triggering was performed if possible. Image analysis of the coronary arteries was performed using dedicated post-processing software (Vitrea FX 7.12; Vital Images, Minnetonka, MN, USA).

### 2.4. CCTA Analysis

Available data on Agatston calcium scores were retrieved, including calcium score percentiles. Elevated CACS was defined as a score > 0. CACS was also scored binary and a score of zero was given in the absence of any plaques or in the presence of wall irregularities or non-calcified plaques. A score of 1 was given if calcified or mixed plaques were present. CCTA scans were analyzed by consensus of experts in the field according to the 17-sement model described by the American Heart Association [14]. Severity of coronary artery stenosis was collected for the main coronary arteries: the left main artery, the left anterior descending artery (LAD), the circumflex artery (Cx), and right coronary artery (RCA). Segments were defined uninterpretable in the case of low contrast enhancement or severe motion artifact as the presence of plaques cannot be excluded due to bad image quality. Interpretable segments were evaluated for stenosis and classified into three categories: no CAD (no plaque or stenosis), non-obstructive (<50% stenosis), and obstructive CAD (≥50% stenosis) according to prior studies [15]. Plaque characteristics were assessed in the presence of plaque and defined as non-calcified, mixed, or calcified [16].

### 2.5. Patient Follow-Up

The start of the study period is defined as the baseline visit at the outpatient cardiology clinic, during which patients presented between 2006 and 2022. Patients were followed from baseline visit until November 2022 for the occurrence of the following major adverse cardiac events: myocardial infarction (defined according to the fourth universal definition of myocardial infarction) [17], coronary revascularization (coronary stent, balloon angioplasty or CABG) > 90 days after CCTA, Transcatheter Aortic Valve Implantation (TAVI), Aortic Valve Replacement (AVR), Mitral Valve Replacement (MVR), and cardiac death. Event data were collected for all patients from the departmental cardiology information system. The cause of death was collected through the civil municipal registry and was classified as malignancy-related death, cardiac death, or death by other causes.

### 2.6. Statistical Analysis

Categorical variables are shown as percentages or frequencies. Continuous variables are expressed as mean ± standard deviation (SD) or median with interquartile range [IQR], depending on whether they follow a normal distribution, which was assessed graphically. To compare the baseline characteristics of continuous variables between HL patients with a CACS of zero or a CACS > 0, the independent Student’s *t*-test or Mann-Whitney U test was used depending on whether the variable followed a normal distribution. Chi-squared tests were used to evaluate differences in binary or categorical variables between groups. The odds ratio (OR) with 95% confidence interval (95% CI) was calculated to assess differences in the prevalence of elevated CACS in HL patients compared to non-cancer control patients and was also calculated adjusted for the prior defined matching factors using logistic regression analysis. The OR with 95% CI was also used to assess the association between radiation doses and an elevated CACS. The reverse Kaplan–Meier method was used to estimate the median follow-up time. A *p*-value of <0.05 was considered as statistically significant. All analyses were performed in STATA version 17.0, (StataCorp 2021, College Station, TX, USA).

## 3. Results

### 3.1. Characteristics of the HL Population

**Figure 1:**
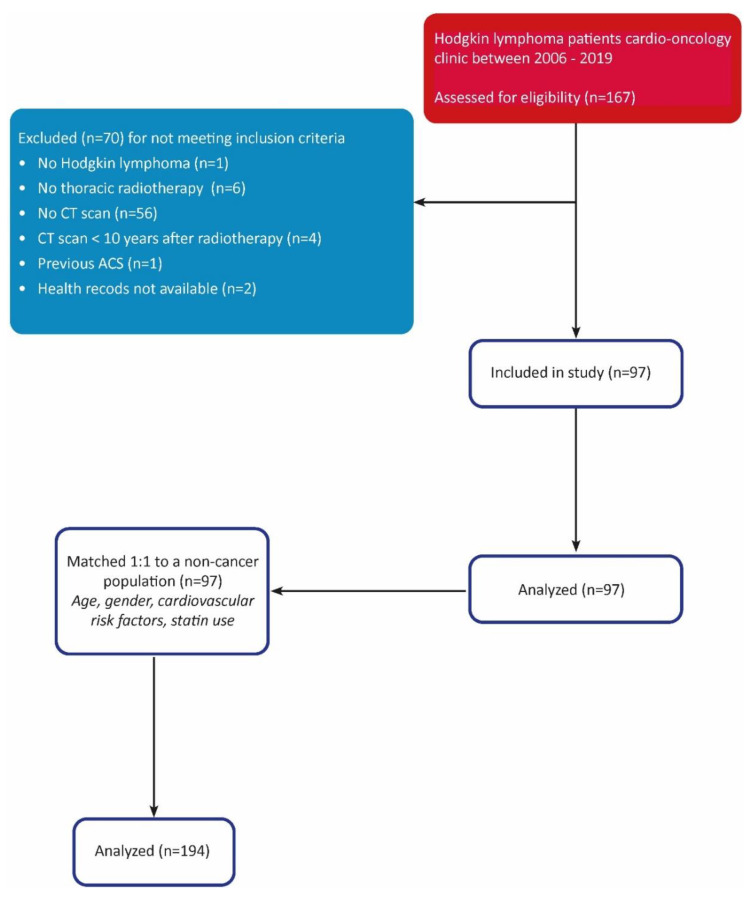
STROBE diagram.

In total, 167 HL patients were screened for eligibility, of whom 70 were excluded. The inclusion process is shown in Figure 1.

The baseline characteristics of the matched study population and of the HL population can be found in Table 1 and Table 2, respectively.

In the HL population, the majority of the patients was female (61.9%) and mean age at HL diagnosis was 25.0 ± 7.6 years. Mantle field irradiation was applied in 34 patients (35.1%), 46 patients (47.4%) were treated with mediastinal radiotherapy, 13 patients (13.4%) with subtotal nodal radiotherapy, and 4 patients (4.1%) were treated with another type of radiotherapy. In addition to radiotherapy, 81 patients (83.5%) were treated with chemotherapy, of whom 66 (84.0%) were treated with an anthracycline containing regimen. Total radiation doses were available for 95 HL patients (97.9%); 74 patients were treated with doses ≥ 35 Gy, and 21 patients with doses < 35 Gy. Median cumulative radiation dose was 36 [35; 40] Gy. No data were available on MHD. Table 3 shows the relationship between these different radiation doses and the presence of elevated CACS more than 10 years after radiotherapeutic treatment. Exposure to radiation doses ≥ 35 Gy was significantly associated with an elevated CACS (OR = 8.8 [95%CI: 2.2–49.6], *p* < 0.001) compared to patients treated with radiation doses < 35 Gy.

### 3.2. CAC Scores Compared to General Population

The 97 HL patients were successfully matched to 97 non-cancer control patients from a large CCTA database according to prior defined matching criteria. In the HL population, 49 patients (50.5%) had an elevated CACS compared to 30.9% (*n* = 30) in the non-cancer population (*p =* 0.005). Patients with HL had a more than two-fold increased prevalence of having CACS > 0 compared to non-cancer patients with an OR of 2.28 [1.22–4.28] (*p* = 0.006). When adjusted for the prior defined matching factors, patients with HL had an OR of 3.05 [1.52–6.17] for a CAC score > 0 compared to the control population (*p* = 0.002).

### 3.3. Agatston Calcium Scores in the Matched Study Population

**Figure 2:**
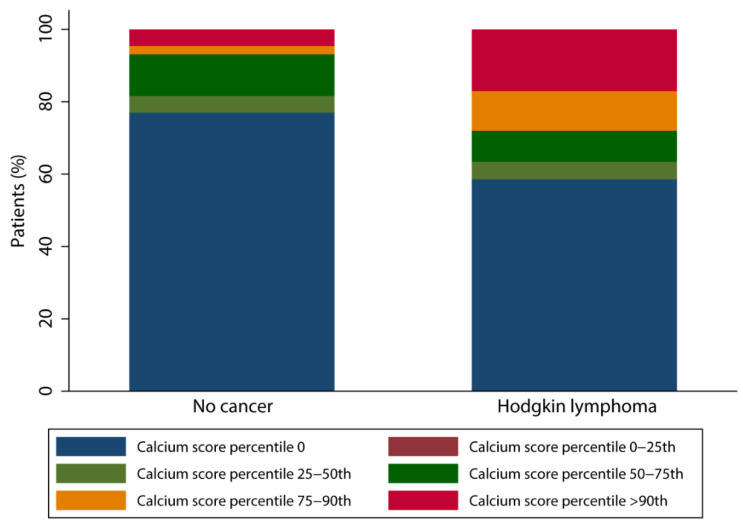
Bar chart of calcium score percentile distribution.

Agatston calcium scores were available for 82 patients (84.5%) in the HL population and these scores ranged from 0 to 1112 with a median score of 0 [p25; p75 = 0;14]. In the non-cancer population, Agatston calcium scores were available for 87 patients (89.7%) and scores ranged between 0 and 751 with a median of 0 [0; 0]. A different distribution pattern of calcium score percentiles corrected for age and gender was found between the two groups. As shown in the Figure 2, the prevalence of a calcium score above the 90th percentile is higher in HL survivors (17.1% vs. 4.6%, *p =* 0.009) and in line, non-cancer patients more often show a calcium score percentile of 0 (77.0% vs. 58.5%, *p* = 0.01).

**Figure 3:**
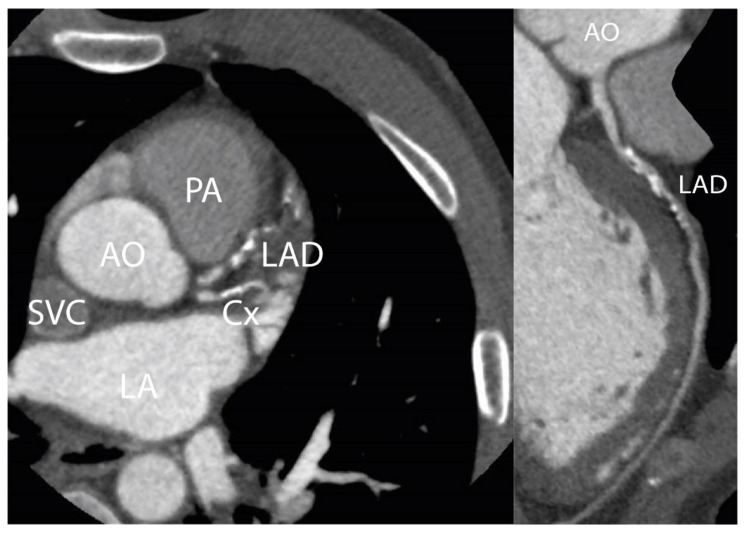
CCTA scan. Figure 3 shows a CCTA scan of a 50-year-old male patient with three-vessel disease 25 years after irradiation for HL. This patient had no cardiovascular risk factors: RR 130/80 mmHg, total cholesterol 3.4 mmol/L, LDL-cholesterol 2.1 mmol/L, no diabetes or history of smoking. A significant stenosis in the mid-LAD is shown. Agatston calcium score was 512, which was >90th percentile corrected for age and gender. AO = aorta; LAD = left anterior descending artery; PA = pulmonary artery; LA = left atrium; Cx = circumflex artery; SVC = superior vena cava.

Figure 3 shows an example of a CCTA scan of a HL survivor with a calcium score > 90th percentile and >50% stenosis of the mid-LAD.

We also performed a sensitivity analysis after excluding all patients without Agatston calcium scores and their matches. This study population consisted of 74 HL patients and 74 non-cancer patients. A CAC score of zero was significantly more prevalent in non-cancer patients compared to HL survivors (77% vs. 61%, *p =* 0.03). The calculated OR for HL patients for having a CACS > 0 was 2.16 [1.00–4.73], *p* = 0.03. When adjusted for the prior defined matching factors, the OR for having a CACS > 0 was 3.42 [1.38–8.50] for HL survivors compared to the control group (*p* = 0.008). In this group, the prevalence of a calcium score above the 90th percentile was also significantly higher in the HL population (18% vs. 4.1%, *p* = 0.008).

### 3.4. Obstructive and Non-Obstructive Stenosis and Plaque Characteristics on CCTA

Five patients did not achieve the target heart rate after preparation and therefore only CACS was performed. Therefore, data on coronary stenosis are available for 94 HL and 95 non-cancer patients. Median heart rate for HL patients was 62.4 [55.1; 71.8] bpm, and for non-cancer patients this was 59.7 [53.1; 68.1] bpm (*p =* 0.3). No significant differences for scan quality were found between the two groups. Non-obstructive coronary artery stenosis was observed in 43 (45.7%) HL patients and in 27 (28.4%) non-cancer patients (*p* = 0.01). No differences in plaque composition were found between HL patients and the control group. Calcified plaques were present in 31.9% of the HL patients and 20.0% of the control patients (*p* = 0.06). For mixed plaques, this was 26.6% vs. 16.8%, respectively (*p* = 0.10), and non-calcified plaques were found in 27.7% of the HL patients vs. 17.9% of the control patients (*p* = 0.11). Obstructive stenosis in any of the coronary arteries was observed in nine (9.6%) HL patients and in four (4.2%) non-cancer patients (*p* = 0.15). Table 4 shows an overview of the presence of non-obstructive and obstructive CAD per Agatston calcium score interval. Non-obstructive CAD in the absence of calcifications (i.e., non-calcified plaque) and a Agatston score ≥ 100 are show a trend towards a significant higher prevalence in HL patients compared to the control population. Time between radiation and CCTA scan is also described for each Agatston score interval and differed significantly (*p* < 0.001). Our observations reveal a higher prevalence of calcium scores > 0 in patients scanned after a longer period post-irradiation, especially in individuals with Agatston calcium scores ≥ 100.

### 3.5. Echocardiography and Valvular Replacement in the HL Population

The majority of the patients (89.7%) had a good left ventricular ejection fraction (LVEF), while the other 10.3% had a moderate LVEF. Twelve patients (12.4%) showed stenosis of the aortic valve, of which one was severe, four moderate, and seven mild. Two patients showed mild stenosis of the mitral valve and one patient moderate stenosis. During follow-up, 12 patients experienced 15 valvular events: eight patients received an AVR, of whom three were combined with MVR, and in four patients a TAVI was performed. A CACS > 0 at baseline was observed in 91.7% of the patients who experienced valve surgery during follow-up. We found a significant association between a CACS > 0 at baseline and a valve procedure during follow-up (*p* = 0.002). Also, all patients who underwent valve surgery had prior radiotherapy doses ≥ 35 Gy (*p* = 0.048).

### 3.6. Cardiovascular Events

During a median follow-up period of 8.5 [5.3; 9.9] years, nine HL patients (9.3%) experienced one or more coronary events: two patients presented with myocardial infarction, two patients underwent CABG, and four patients underwent percutaneous revascularization. Two patients, of whom one had a previous percutaneous revascularization, died due to a cardiac cause. An overview of CAC scores, CAC percentiles, and findings on CCTA for all HL survivors that experienced one or more coronary events during follow-up is presented in Supplemental Table S1. Interestingly, two HL patients (2.1%) who experienced a coronary event < 10 years after CCTA had a CACS of zero at baseline CCTA. Detailed patient case descriptions of two patients can be found in Appendix A. None of the patients in the non-cancer population experienced a major adverse cardiac event during follow-up. 

## 4. Discussion

To our knowledge, although prior studies have focused on premature CAD and risk for cardiovascular events in HL survivors, this is the first study conducted to assess differences in the presence of CAC between HL patients treated with radiotherapy compared to a non-cancer control group matched on age, gender, and cardiovascular risk factors. The key finding of this study is that HL survivors have a higher prevalence of a CACS > 0 compared to a matched non-cancer population. In particular, thoracic radiotherapy doses ≥ 35 Gy were associated with a significantly increased prevalence of an elevated CACS. Our results also revealed that despite a CACS of zero, patients in the population of HL survivors were at increased risk for cardiovascular events, with an event rate of 2.1% during follow-up.

### 4.1. CACS in HL Patients

Several studies have described the association between CACS and CAD in HL survivors. Andersen et al. [18] studied 47 HL survivors treated with mediastinal radiotherapy and assessed CACS in relation to CAD confirmed by invasive coronary angiography. The authors found that 83% of the patients presented with a CACS > 0. The current study shows that HL survivors have a higher prevalence of CACS compared to a matched cohort, and a calcium score > 90th percentile was disproportionate to what was expected, based on age and risk factors. In addition, in HL survivors who were treated with mediastinal radiotherapy, significant three-vessel disease has been described despite a CACS of zero [19]. Usually, a CACS of zero is associated with very low risk of coronary stenosis; however, radiation may induce the development of other plaque compositions, leading to non-calcified plaques that are not detected by calcium scoring. In populations treated with thoracic radiotherapy, performing a CCTA scan instead of calcium scoring may be preferred to detect obstructive stenosis.

### 4.2. CACS for Risk Assessment, Monitoring, and Prevention

Measuring CACS has become a widely validated method for coronary risk assessment. A scientific statement from the American Heart Association (AHA) [20] describes a very low risk of 0.1%/year associated with a CACS of zero for a cardiovascular event in the following five years [21]. Various studies have assessed the predictive value of a CACS of zero on future CAD showing a very low annual event rate of 0–0.9 per 100 patients during follow-up periods with a maximum of seven years [22,23,24,25,26,27] In our study, 4.2% of the HL patients who had a CACS of zero experienced a coronary event within 8.5 years after CCTA. Therefore, the warranty period for a CACS of zero seems to be different in HL survivors compared to the general population, as the natural history of radiotherapy related vasculopathy is different from atherosclerosis and may accelerate rapidly. In breast cancer patients treated with radiotherapy, a baseline CAC higher than zero was associated with progression of CAC after irradiation, and this progression was observed more often in left-sided breast cancer compared to right-sided breast cancer [28,29]. Progression from a CACS of zero to a CAC score > 0 after breast cancer irradiation occurred in >5% of the breast cancer patients after two years [28]. As studies have described increased calcium scores, as well as soft plaques leading to significant stenosis after radiotherapy, risks associated with (high) Agatston scores could be elevated compared to the general population as these plaques possibly consist of more extensive non-calcified parts. In our study, the risk of CAD increased with increasing radiation doses, in line with observations in prior studies [30,31].

If we correlate the mean age and CACS of HL patients to the estimated arterial ages based on CACS described by McClelland et al. [32], arterial age exceeds biological age in the majority of the HL population. Moreover, this correlation is presumably underestimated due to a history of radiotherapy.

According to the expert consensus statements of the International Cardio-Oncology Society (ICOS) and Society of Cardiovascular Computed Tomography (SCCT) [10,33], the atherosclerotic cardiovascular disease (ASCVD) risk should be calculated in asymptomatic cancer survivors. Depending on the calculated risk, it is recommended to perform a CAC scan or initiate treatment with cardiac medication. For very low ASCVD risk of <5%, the guidelines only recommend re-evaluating risk scores after one year [33]. Screening guidelines also recommend to annually review available CT imaging for atherosclerotic disease in patients irradiated in the thoracic region [33]. Evaluation of CAD with CAC, CCTA, or functional stress testing for ischemia is recommended at five-year intervals, which is in correspondence with the most recent ESC guidelines [34]. However, it is not specified whether CACS or CCTA should be performed. Prior research in HL survivors treated with radiotherapy has shown that CCTA is the leading imaging modality in detecting early radiation-induced CAD [7,8,35]. According to the most recent SCCT consensus statement, CCTA should be considered as an initial cardiac imaging modality to assess radiation-induced CAD in symptomatic patients without prior CAD, whereas in asymptomatic patients, the authors recommend CAC scoring as the preferred modality [10]. Our study shows that performing CCTA in asymptomatic patients after radiation may be preferred as non-calcified plaques can be detected that lead to severe atherosclerotic disease [19]. Atherosclerotic plaques are different from conventional CAD, with a greater degree of fibrosis and a lower lipid or calcium burden [9]. Our results support these findings, showing that despite a CACS of zero, HL survivors were at increased risk for cardiovascular events. Although no significant differences were found in the current study, most probably due to small sample size, we observed a trend in a higher prevalence of non-calcified plaques in patients after radiation, and therefore larger cohorts are needed.

### 4.3. Initiation of Preventive Medication

In the general population, the CAC-Data Reporting System (CAC-DRS) is used to determine the importance of initiating preventive cardiovascular treatment. We describe two patients in our supplemental material who at young age already had wall irregularities up to 30%, high LDL-cholesterol, and 30–50% stenotic plaques. In our population, HL patients with a CACS of zero still seem to have an increased risk of future cardiovascular events, and timely treatment with statins may be considered in this patient population. Moreover, it would be interesting to correlate Agatston calcium scores to arterial age in populations treated with irradiation to guide initiation of treatment [32].

### 4.4. Study Limitations

First, this is a single-center matched cross-sectional study, and therefore no temporal relation between thoracic radiation and CAC could be assessed. Furthermore, a relatively small sample size of 97 cases was available. Also, as asymptomatic HL survivors were matched to a symptomatic control group with complaints of chest pain, this could underestimate the increased risk of HL survivors for the prevalence of an increased CACS. Moreover, the small number of patients in each category of the radiotherapy location made it unfeasible to assess the effect of irradiated fields on CACS or significant CAD. Furthermore, the Agatston scores and calcium score percentiles were not available for the entire study population, and quantitative plaque analysis was not performed.

## 5. Conclusions

HL survivors treated with radiotherapy have a significantly higher prevalence of a CACS > 0 compared to a non-cancer matched control population. In particular, radiotherapy doses ≥ 35 Gy were associated with a significantly higher prevalence of an elevated CACS. Interestingly, HL patients with a CACS of zero still have an increased risk of future cardiovascular events compared to healthy study populations, possibly due to an increased incidence of non-calcified plaques and rapid progression of atherosclerosis in the coronary arteries following irradiation. Therefore, performing only a calcium score may not be sufficient in this population, and CCTA is crucial to exclude significant plaque burden and CAD.

## Figures and Tables

**Table 1:** Baseline characteristics of the matched study population.

	Total (*n* = 194)	No Cancer (*n* = 97)	Cancer (*n* = 97)	*p*-Value
Female sex	120 (61.9%)	60 (61.9%)	60 (61.9%)	1.00
Age during CT scan, years	45.5 ± 10.0	45.5 ± 10.3	45.5 ± 9.9	0.98
Cardiovascular risk factors				
History of hypertension	26 (13.4%)	13 (13.4%)	13 (13.4%)	1.00
History of hypercholesterolemia	104 (53.6%)	52 (53.6%)	52 (53.6%)	1.00
History of smoking	16 (8.2%)	8 (8.2%)	8 (8.2%)	1.00
Statin use	10 (5.2%)	5 (5.2%)	5 (5.2%)	1.00

**Table 2:** Baseline characteristics of the HL population.

	Total (*n* = 97)	CACS = 0 (*n* = 48)	CACS > 0 (*n* = 49)	*p*-Value
Female sex	60 (61.9%)	29 (60.4%)	31 (63.3%)	0.77
Age at diagnosis, years	25.0 ± 7.6	24.4 ± 8.1	25.6 ± 7.0	0.17
Age at CT scan, years	45.5 ± 9.9	41.0 ± 8.8	49.9 ± 9.0	<0.001
Year of radiotherapy treatment initiation	1994 [1987; 2000]	2000 [1992; 2001]	1989 [1986; 1995]	0.42
Cardiovascular risk factors				
History of hypertension	13 (13.4%)	2 (4.2%)	11 (22.4%)	0.008
History of hypercholesterolemia	52 (53.6%)	21 (43.8%)	31 (63.3%)	0.054
History of smoking	8 (8.2%)	5 (10.4%)	3 (6.1%)	0.44
Statin use	5 (5.2%)	1 (2.1%)	4 (8.2%)	0.18
Stage of Hodgkin lymphoma				0.52
I–II	72 (74.2%)	37 (77.1%)	35 (71.4%)	
III–IV	25 (25.8%)	11 (22.9%)	14 (28.6%)	
Location of radiotherapy				0.018
Mantle	34 (35.1%)	10 (20.8%)	24 (49.0%)	
Mediastinal	46 (47.4%)	30 (62.5%)	16 (32.7%)	
Subtotal	13 (13.4%)	6 (12.5%)	7 (14.3%)	
Other	4 (4.1%)	2 (4.2%)	2 (4.1%)	
Radiotherapy dose, Gy	36.0 [35.0; 40.0]	36.0 [30.0; 40.0]	36.0 [36.0; 40.0]	0.063
Treated with chemotherapy	81 (83.5%)	42 (87.5%)	39 (79.6%)	0.29
Chemotherapeutic regimen				0.027
ABVD	27 (27.8%)	16 (33.3%)	11 (22.4%)	
MOPP/ABV	24 (24.7%)	10 (20.8%)	14 (28.6%)	
EBVP	9 (9.3%)	7 (14.6%)	2 (4.1%)	
BEACOPP	6 (6.2%)	5 (10.4%)	1 (2.0%)	
MOPP	12 (12.4%)	2 (4.2%)	10 (20.4%)	
Other	3 (3.1%)	2 (4.2%)	1 (2.0%)	
Treatment with anthracyclines	68 (70.1%)	39 (81.3%)	29 (59.2%)	
Doxorubicine	60 (61.9%)	32 (66.7%)	28 (57.1%)	
Epirubicine	8 (8.2%)	7 (14.6%)	1 (2.0%)	
Laboratory values				
Hb, mmol/L	8.8 ± 0.8	8.8 ± 0.9	8.7 ± 0.8	0.53
Leukocytes, ×10^9^/L	6.8 ± 1.8	6.6 ± 1.8	7.1 ± 1.8	0.19
LDL, mmol/L	3.1 ± 1.0	3.1 ± 1.1	3.1 ± 1.0	0.88
Total cholesterol, mmol/L	5.3 ± 1.0	5.2 ± 0.9	5.4 ± 1.1	0.35
Creatinine μmol/L	75.1 ± 13.7	76.5 ± 14.9	73.9 ± 12.5	0.36
Left ventricular function				0.97
Good	87 (89.7%)	43 (89.6%)	44 (89.8%)	
Moderate	10 (10.3%)	5 (10.4%)	5 (10.2%)	
Poor	0	0	0	
Aortic stenosis	12 (12.4%)	2 (4.2%)	10 (20.4%)	0.015
Mild	7 (58.3%)	2 (4.2%)	5 (10.2%)	
Moderate	4 (33.3%)	0	4 (8.2%)	
Severe	1 (8.3%)	0	1 (2.0%)	
Mitral stenosis	3 (3.1%)	0	3 (6.1%)	0.08
Mild	2 (66.7%)	0	2 (4.1%)	
Moderate	1 (33.3%)	0	1 (2.0%)	
Severe	0	0	0	

Table 2 shows the baseline characteristics of the HL population. Differences are shown for patients with a CACS of zero vs. patients with a CACS > 0. Laboratory values correspond to a time point between one year before and one year after baseline visit. Good LV function was defined as ≥50%, moderate as 30–49%, and poor as <30%. Gy = gray; ABVD = Adriamycin, Bleomycin, Vinblastine, Dacarbazine; MOPP/ABV = Mustargen, Oncovin, Procarbazine, Prednisone, Adriamycin, Bleomycin, Vinblastine; EBVP = Epirubicine, Bleomycin, Vinblastine, Prednisone; BEACOPP = Bleomycin, Etoposide, Adriamycin, Cyclophosphamide, Oncovin, Procarbazine, Prednisone; MOPP = Mustargen, Oncovin, Procarbazine, Prednisone; Hb = hemoglobin; LDL = low-density lipoprotein; ACS = acute coronary syndrome; PCI = percutaneous coronary intervention. Reference values: Hb 8.5–11 mmol/L (m), 7.5–10.0 (f), leukocytes 4.0–10.0, LDL < 3.0, total cholesterol < 5.0, creatinine 64–104 (m), 49–90 (f). The differences between the two groups were compared using chi-squared tests for binary and categorical variables, unpaired *t*-tests for normally distributed continuous variables, and Mann–Whitney U-tests for non-normally distributed continuous data.

**Table 3:** Association between radiation dose and calcium score in HL patients.

	CACS > 0 (*n* = 47)	CACS = 0 (*n* = 48)	OR [95%CI]	*p*-Value
Radiation dose			8.8 [2.2–49.6]	<0.001
<35 Gy	3 (6.4%)	18 (37.5%)		
≥35 Gy	44 (93.6%)	30 (60.5%)		
Radiation dose 15–34 Gy + doxorubicin ≥ 100 mg/m^2^			0.04 [0.0–0.3]	<0.001
Yes	1 (2.1%)	16 (33.3%)		
No	46 (97.9%)	32 (66.7%)		

Table 3 shows the association between different treatment radiation doses and the presence of an elevated calcium score in the HL population. Radiation doses ≥ 35 Gy are significantly associated with a CACS > 0. Combined treatment with radiation doses 15–34 Gy with doxorubicin doses ≥ 100 mg/m^2^ does not show this association compared to patients treated with radiotherapy doses ≥ 35 Gy alone or in combination with doxorubicin treatment. One patient in the comparison group was treated with 15–34 Gy irradiation combined with doxorubicin < 100 mg/m^2^. Groups are compared using the chi-squared test and the odds ratios were calculated. CACS = coronary artery calcium score; Gy = Gray; OR = odds ratio.

**Table 4:** Agatston calcium score and presence of coronary artery stenosis.

Agatston Calcium Score	Time between Radiation and CCTA, years	HL Population (*n* = 82)	Control Population (*n* = 87)	*p*-Value
0	14.2 [11.8–20.7]	48 (58.5%)	67 (77.0%)	0.005
Non-obstructive	15.0 [13.5–20.7]	8 (9.8%)	4 (4.6%)	0.06
Obstructive	20.7 [20.7–20.7]	1 (1.2%)	1 (1.1%)	0.81
1–99	24.3 [18.2–25.3]	23 (28.0%)	16 (18.4%)	0.21
Non-obstructive	24.6 [19.0–25.3]	17 (20.7%)	13 (14.9%)	0.59
Obstructive	18.2 [17.2–19.1]	3 (3.7%)	1 (1.1%)	0.49
≥100	24.3 [22.3–25.9]	11 (13.4%)	4 (4.6%)	0.06
Non-obstructive	23.3 [21.4–25.9]	7 (8.5%)	3 (3.4%)	0.68
Obstructive	24.8 [23.8–26.3]	4 (4.9%)	1 (1.1%)	0.68

Table 4 shows the relation between Agatston calcium scores, time between radiation and CCTA scan, and the presence of non-obstructive (<50%) and obstructive (≥50%) coronary artery disease (CAD) in any of the coronary arteries. Per Agatston score interval, the number of corresponding patients is shown as well as the percentage of those patients with non-obstructive or obstructive CAD. Agatston calcium scores were available for 82 HL patients and 87 control patients.

**Figure A1:**
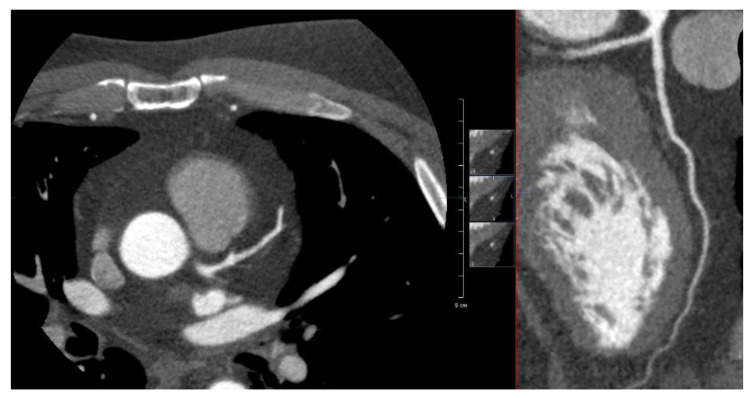
Figure A1 shows an axial view and multiplanar reconstruction view of the LAD during CCTA in 2012 without any signs of stenosis in the LAD.

**Figure A2:**
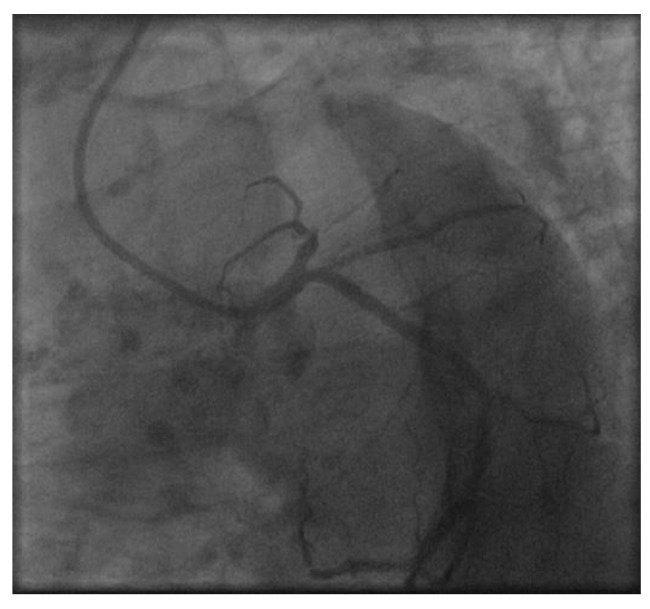
Figure A2 shows the LAD during cardiac catheterization in 2020 with a subtotal stenosis in the proximal LAD.

**Figure A3:**
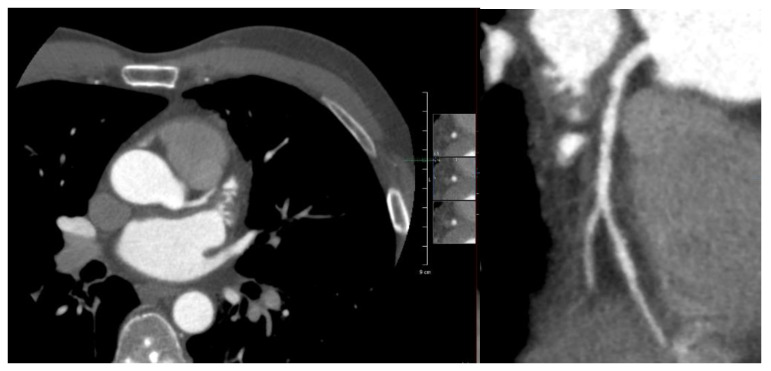
Figure A3 shows an axial and multiplanar reconstruction view of the LAD in 2012. The proximal LAD shows non-calcified plaques, graded as not significant.

**Figure A4:**
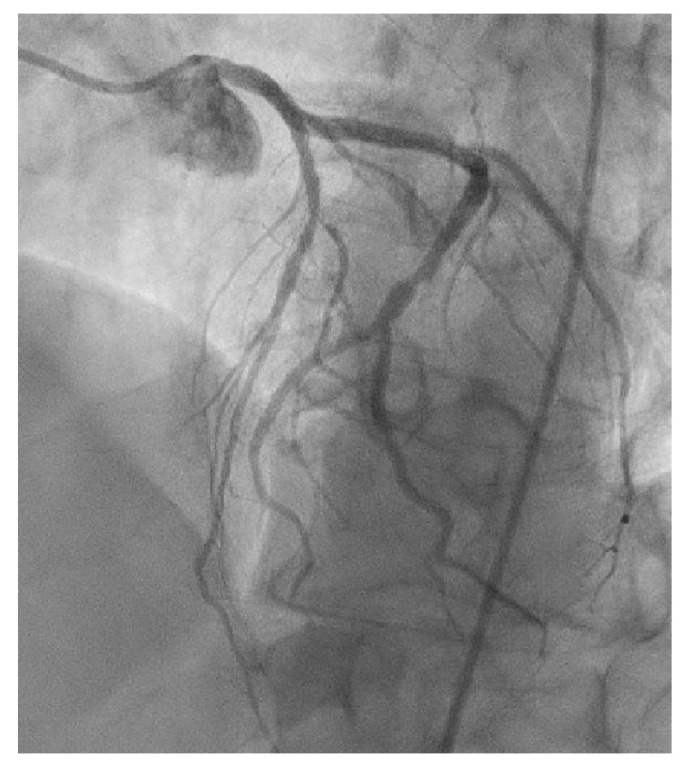
Figure A4 shows the significant stenosis of the mid-LAD with occlusion of the third diagonal branch during cardiac catheterization in 2016.

## Data Availability

The data underlying this article will be shared on reasonable request to the corresponding author.

## Supplementary Materials

The following supporting information can be downloaded at: https://www.mdpi.com/article/10.3390/cancers15245831/s1, Table S1. Event descriptions of HL survivors.

## Appendix A. Patient Case Descriptions

### Appendix A.1. Patient A. Male, 46 Years

This case describes a 46-year-old male patient who was diagnosed with HL in 2000. He was treated with chemotherapy (8× Adriamycin/Bleomycin/Vinblastine/Dacarbazine) and mediastinal radiotherapy with a maximum radiation dose of 24 Gy. In 2012, he was referred to the outpatient cardio-oncology clinic for regular screening according to the Dutch National Survivorship Care Pathway. Cardiovascular risk factors were an elevated total cholesterol as well as LDL-cholesterol levels (7.42 mmol/L and 3.79 mmol/L, respectively) and BMI was 33.8. He had no cardiac complaints, and his ECG and exercise stress test showed no abnormalities. Echocardiogram showed a good left ventricular function without any signs of valve abnormalities. The Agatston calcium score was zero, and on CCTA only wall irregularities < 30% were seen in the LAD and LCx (Figure A1). In 2020, he presented at the emergency department with typical anginal chest pain for the last few months. ECG showed ST-segment elevation anteriorly. The LAD showed proximal subtotal stenosis for which primary PCI was performed with one drug-eluting stent (DES) (Figure A2). Furthermore, 70% stenosis of the mid-RCA, 60% stenosis of the mid-LAD, and 60% stenosis of the right descending posterior branch (RDP) were observed.

### Appendix A.2. Patient B. Male, 45 Years

This case refers to a 45-year-old male patient who was diagnosed with HL in 2001. He was treated with chemotherapy (6× Adriamycin/Bleomycin/Vinblastine/Dacarbazine) and radiotherapy on bulky tumor areas with a maximum radiation dose of 40 Gy. In 2012, he was referred to the outpatient cardio-oncology clinic for regular screening according to the Dutch National Survivorship Care Pathway. Except for active smoking, there were no cardiovascular risk factors. He had no cardiac complaints, and his ECG and exercise stress test showed no abnormalities. On echocardiography, a good left ventricular function was seen without any signs of valve disease. The Agatston calcium score was 1 (LAD), and on CCTA non-calcified plaques were seen in the proximal LAD (30–50%) and in the mid-LCx (30%) and mid-RCA (30%) (Figure A3). In 2016, patient B presented at the emergency department with an out-of-hospital cardiac arrest caused by ST-elevation myocardial infarction. ECG showed elevated ST-segments anteriorly. Primary PCI was performed with one DES in the mid-LAD, which showed 60–70% stenosis and chronic total occlusion of the third diagonal branch (Figure A4). Furthermore, 50% stenosis of the RDP and 50–60% stenosis of the ostium of a non-dominant RCA were observed.
