# Supplementary material for: Relation between Coronary Artery Calcium Score and Cardiovascular Events in Hodgkin Lymphoma Survivors: A Cross-Sectional Matched Cohort Study
Source: Cancers (Basel). 2023 Dec 13;15(24):5831. doi: 10.3390/cancers15245831 (PMC10742169; doi:10.3390/cancers15245831)
Supplement: Supplementary file 1 [file cancers-15-05831-s001.zip › cancers-2708950-supplementary.pdf]

Table S1. Event descriptions of HL survivors.

Patient	Sex	Age	Agatston Score	CCTA Findings	Follow-up
1	M	46	0	07-2012: wall irregularities <30% in LAD and LCx	12-2020: acute ST-elevation myocardial infarction. PCI with stenting of the LAD
2	F	62	472 (>90th percentile)	09-2012: calcified and mixed plaques in all coronary arteries <50%	03-2016: PCI with stenting of the RCA, RCx and LAD
3	F	42	NR	07-2010: calcified spot <30% in LM. Wall irregularities <30% in LAD and LCx	12-2018: PCI with stenting of the LM, LAD and Cx
4	M	45	1 (25-50th percentile)	09-2012: non-calcified plaques <50% in the proximal LAD, mid RCA and mid LCx. Wall irregularities in all coronary arteries	03-2016: out of hospital cardiac arrest caused by ST-elevation myocardial infarction. PCI with stenting of the LAD
5	M	54	438 (>90th percentile)	12-2011: mixed plaque >70% in MO. Mixed plaques <50% In LAD and LCx. Wall irregularities <30% in all coronary arteries	03-2017: cardiac death as a result of S. Bovis endocarditis of mechanical AVR and MVR
6	M	48	0	03-2012: wall irregularities <30% in the LM, LAD and LCx	09-2016: PCI with stenting of the RCA
7	M	55	1112 (>90th percentile)	09-2012: calcified plaques in all coronary arteries, grade of stenosis unavailable due to poor scan quality	04-2013: CABG LIMA-D-LAD and RIMA-MO
8	M	40	14 (50-75th percentile)	05-2011: non calcified plaques <50% in D2 and PL. Mixed plaque 30% in proximal RCA. Wall irregularities <30% in all coronary arteries	04-2018: PCI with stenting of the LAD
9	M	49	132 (75-90th percentile)	12-2011: non-calcified, mixed and calcified plaques with <50% in LAD and LCx. Wall irregularities in all coronary arteries	08-2017: CABG LIMA-RI-PLCx

RCA = right coronary artery; LM = left main coronary artery; LAD = left anterior descending artery; LCx = circumflex artery; PCI = percutaneous coronary intervention; CABG = coronary artery bypass grafting; LIMA = left internal mammary artery; RIMA = right internal mammary artery; MO = obtuse marginal artery; RCx = circumflex artery; AVR = aortic valve replacement; MVR = mitral valve replacement; D = diagonal branch artery; PL(Cx) = posterolateral artery; RI = ramus intermedius branch; NR = not reported.
